# 6,8-Di-*tert*-butyl-3-(4-nitro­phen­yl)-2*H*-chromen-2-one

**DOI:** 10.1107/S1600536810002321

**Published:** 2010-01-30

**Authors:** XiaoFeng Zhou

**Affiliations:** a College of Transportation, Southeast University, Nanjing 210096, People’s Republic of China, and Department of Physics, Southeast University, Nanjing 210096, People’s Republic of China

## Abstract

The title compound, C_23_H_25_NO_4_, was synthesized by the reaction of 2-(4-nitro­phen­yl)acetonitrile and 3,5-di-*tert*-butyl-2-hydroxy­benzaldehyde. The dihedral angle formed by the benzene ring and the mean plane through the benzopyran­one ring system is 35.57 (5)°. The nitro group is almost coplanar with the attached benzene ring [dihedral angle = 5.19 (15)°]. The crystal packing is stabilized by an inter­molecular C—H⋯O hydrogen-bond inter­action.

## Related literature

For the applications and biological activity of coumarin deriv­atives, see: Tian *et al.* (2000 ▶); Fun *et al.* (2009 ▶).
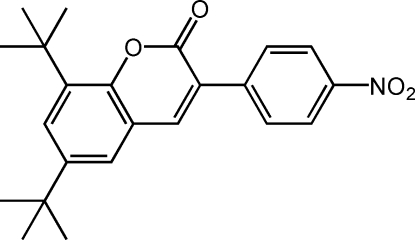


## Experimental

### 

#### Crystal data

C_23_H_25_NO_4_
*M* *_r_* = 379.44
Orthorhombic, 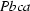

*a* = 14.6463 (13) Å
*b* = 11.8634 (10) Å
*c* = 23.604 (2) Å
*V* = 4101.3 (6) Å^3^
*Z* = 8
Mo *K*α radiation
μ = 0.08 mm^−1^
*T* = 293 K
0.20 × 0.20 × 0.10 mm

#### Data collection

Bruker SMART CCD area-detector diffractometer
Absorption correction: multi-scan (*SADABS*; Bruker, 2000 ▶) *T*_min_ = 0.982, *T*_max_ = 1.000
33748 measured reflections
4736 independent reflections
2809 reflections with *I* > 2σ(*I*)
*R*_int_ = 0.053

#### Refinement

*R*[*F* ^2^ > 2σ(*F* ^2^)] = 0.046
*wR*(*F* ^2^) = 0.140
*S* = 1.01
4736 reflections
254 parameters
H-atom parameters constrained
Δρ_max_ = 0.19 e Å^−3^
Δρ_min_ = −0.18 e Å^−3^

### 

Data collection: *SMART* (Bruker, 2000 ▶); cell refinement: *SAINT* (Bruker, 2000 ▶); data reduction: *SAINT*; program(s) used to solve structure: *SHELXS97* (Sheldrick, 2008 ▶); program(s) used to refine structure: *SHELXL97* (Sheldrick, 2008 ▶); molecular graphics: *SHELXTL* (Sheldrick, 2008 ▶); software used to prepare material for publication: *SHELXL97*.

## Supplementary Material

Crystal structure: contains datablocks I, global. DOI: 10.1107/S1600536810002321/rz2409sup1.cif

Structure factors: contains datablocks I. DOI: 10.1107/S1600536810002321/rz2409Isup2.hkl

Additional supplementary materials:  crystallographic information; 3D view; checkCIF report

## Figures and Tables

**Table 1:** Hydrogen-bond geometry (Å, °)

*D*—H⋯*A*	*D*—H	H⋯*A*	*D*⋯*A*	*D*—H⋯*A*
C6—H6*A*⋯O2^i^	0.93	2.55	3.409 (3)	154

Symmetry code: (i) 
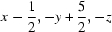
.

**Fig. 1.**
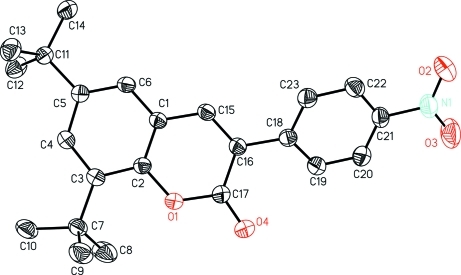
The molecular structure of the title compound, showing the atomic numbering scheme. Displacement ellipsoids are drawn at the 30% probability level. Hydrogen atoms are omitted for clarity.

## supplementary crystallographic information

### Comment

Coumarin (1-benzopyran-2-one) derivatives are a class of important organic compounds which have been found to be very useful in many applications as nonlinear optical materials, laser dyes, fluorescence materials, photorefractive materials, luminescence materials and as intermediates for drug synthesis (Tian *et al.*, 2000). In addition, many natural coumarins possess a wide range of biological activities such as antifungal, antioxidant and antitumor activities (Fun *et al.* 2009). Herein the synthesis and crystal structure of the title compound is reported.

The molecular structure and atom-numbering scheme of the title compound are shown in Fig. 1. The C15—C16 bond is 1.347 (2) Å, which corresponds well to a typical C═C double bond. In addition, the C18—C16—C15 and C16—C15—C1 bond angles are almost equal (122.24 (15) and 122.66 (16)° respectively). The coumarin ring system, consisting of atoms C15, C16, C17, O1, O4 and C1—C6, is almost planar with a maximum deviation from the least-squares plane of 0.0442 (16) Å for atom O4. The phenyl ring attached at the C16 atom is twisted by a dihedral angle of 35.57 (5)°. The nitro group is slightly rotated about the C—N bond by 5.19 (15)°. The crystal packing is stabilized by an intermolecular C—H···O hydrogen bond (Table 1).

### Experimental

2-(4-Nitrophenyl)acetonitrile (486 mg, 3 mmol) and 3,5-di-tert-butyl-2-hydroxybenzaldehyde (703 mg, 3 mmol) were dissolved in ethanol (20 ml) in a 50-ml round-bottom flask equipped with a magnetic stir bar and a water-cooled reflux condenser under nitrogen. The mixture was heated to reflux for 15 minutes, then three drops of piperidine and acetic acid were added. The solution was allowed to reflux for 4 h under nitrogen. After cooling, the reaction mixture was evaporated to dryness using a rotary evaporator to yield a yellow solid. The title compound was recrystallized from ethanol. Single crystals suitable for X-ray analysis were obtained by slow evaporation of an ethanol solution at room temperature.

### Refinement

All H atoms were located geometrically and treated as riding atoms, with C—H = 0.93–0.96 Å, and with *U*_iso_(H) = 1.2 *U*_eq_(C) or 1.5 *U*_eq_(C) for methyl H atoms.

### Crystal data

**Table:**

C_23_H_25_NO_4_	*F*(000) = 1616
*M**_r_* = 379.44	*D*_x_ = 1.229 Mg m^−^^3^
Orthorhombic, *P**b**c**a*	Mo *K*α radiation, λ = 0.71073 Å
Hall symbol: -P 2ac 2ab	Cell parameters from 5698 reflections
*a* = 14.6463 (13) Å	θ = 3.1–27.3°
*b* = 11.8634 (10) Å	µ = 0.08 mm^−^^1^
*c* = 23.604 (2) Å	*T* = 293 K
*V* = 4101.3 (6) Å^3^	Block, yellow
*Z* = 8	0.20 × 0.20 × 0.10 mm

### Data collection

**Table:**

Bruker SMART CCD area-detector diffractometer	4736 independent reflections
Radiation source: fine-focus sealed tube	2809 reflections with *I* > 2σ(*I*)
graphite	*R*_int_ = 0.053
Detector resolution: 13.6612 pixels mm^-1^	θ_max_ = 27.5°, θ_min_ = 2.2°
phi and ω scans	*h* = −19→18
Absorption correction: multi-scan (*SADABS*; Bruker,2000)	*k* = −15→15
*T*_min_ = 0.982, *T*_max_ = 1.000	*l* = −30→26
33748 measured reflections	

### Refinement

**Table:**

Refinement on *F*^2^	Secondary atom site location: difference Fourier map
Least-squares matrix: full	Hydrogen site location: inferred from neighbouring sites
*R*[*F*^2^ > 2σ(*F*^2^)] = 0.046	H-atom parameters constrained
*wR*(*F*^2^) = 0.140	*w* = 1/[σ^2^(*F*_o_^2^) + (0.0615*P*)^2^ + 0.7021*P*] where *P* = (*F*_o_^2^ + 2*F*_c_^2^)/3
*S* = 1.01	(Δ/σ)_max_ < 0.001
4736 reflections	Δρ_max_ = 0.19 e Å^−^^3^
254 parameters	Δρ_min_ = −0.18 e Å^−^^3^
0 restraints	Extinction correction: *SHELXL97* (Sheldrick, 2008), Fc^*^=kFc[1+0.001xFc^2^λ^3^/sin(2θ)]^-1/4^
Primary atom site location: structure-invariant direct methods	Extinction coefficient: 0.0021 (4)

### Special details

**Table:**

Geometry. All e.s.d.'s (except the e.s.d. in the dihedral angle between two l.s. planes) are estimated using the full covariance matrix. The cell e.s.d.'s are taken into account individually in the estimation of e.s.d.'s in distances, angles and torsion angles; correlations between e.s.d.'s in cell parameters are only used when they are defined by crystal symmetry. An approximate (isotropic) treatment of cell e.s.d.'s is used for estimating e.s.d.'s involving l.s. planes.
Refinement. Refinement of *F*^2^ against ALL reflections. The weighted *R*-factor *wR* and goodness of fit *S* are based on *F*^2^, conventional *R*-factors *R* are based on *F*, with *F* set to zero for negative *F*^2^. The threshold expression of *F*^2^ > σ(*F*^2^) is used only for calculating *R*-factors(gt) *etc*. and is not relevant to the choice of reflections for refinement. *R*-factors based on *F*^2^ are statistically about twice as large as those based on *F*, and *R*- factors based on ALL data will be even larger.

### Fractional atomic coordinates and isotropic or equivalent isotropic displacement parameters (Å^2^)

**Table:**

	*x*	*y*	*z*	*U*_iso_*/*U*_eq_	
O1	0.14469 (8)	0.87691 (10)	0.19264 (5)	0.0519 (3)	
C1	0.04378 (11)	0.96286 (14)	0.12603 (7)	0.0423 (4)	
C2	0.05769 (11)	0.88906 (14)	0.17099 (7)	0.0425 (4)	
N1	0.50655 (12)	1.22755 (18)	0.01632 (8)	0.0709 (5)	
O2	0.49270 (12)	1.31945 (18)	−0.00487 (8)	0.1067 (6)	
C3	−0.01328 (11)	0.82746 (14)	0.19572 (7)	0.0434 (4)	
O3	0.58048 (12)	1.18104 (17)	0.01503 (9)	0.1104 (7)	
C4	−0.09936 (11)	0.84849 (14)	0.17326 (7)	0.0459 (4)	
H4A	−0.1486	0.8107	0.1893	0.055*	
C5	−0.11757 (11)	0.92252 (14)	0.12817 (7)	0.0433 (4)	
C6	−0.04451 (11)	0.97845 (14)	0.10493 (7)	0.0452 (4)	
H6A	−0.0539	1.0274	0.0747	0.054*	
C7	0.00116 (12)	0.74480 (15)	0.24487 (8)	0.0503 (4)	
C8	0.07354 (17)	0.65680 (19)	0.22987 (11)	0.0861 (7)	
H8A	0.1299	0.6940	0.2208	0.129*	
H8B	0.0534	0.6137	0.1978	0.129*	
H8C	0.0828	0.6075	0.2616	0.129*	
C9	0.02893 (15)	0.8108 (2)	0.29770 (8)	0.0726 (6)	
H9A	0.0843	0.8516	0.2902	0.109*	
H9B	0.0386	0.7595	0.3286	0.109*	
H9C	−0.0187	0.8630	0.3075	0.109*	
C10	−0.08682 (14)	0.68095 (18)	0.25937 (9)	0.0715 (6)	
H10A	−0.1062	0.6380	0.2271	0.107*	
H10B	−0.1337	0.7338	0.2695	0.107*	
H10C	−0.0757	0.6310	0.2906	0.107*	
C11	−0.21566 (11)	0.94186 (15)	0.10840 (7)	0.0475 (4)	
C12	−0.26345 (13)	0.82947 (18)	0.09799 (10)	0.0705 (6)	
H12A	−0.2316	0.7886	0.0690	0.106*	
H12B	−0.3252	0.8430	0.0861	0.106*	
H12C	−0.2637	0.7862	0.1323	0.106*	
C13	−0.26683 (13)	1.00688 (19)	0.15457 (9)	0.0664 (6)	
H13A	−0.2371	1.0778	0.1611	0.100*	
H13B	−0.2669	0.9637	0.1890	0.100*	
H13C	−0.3286	1.0199	0.1426	0.100*	
C14	−0.21945 (13)	1.0105 (2)	0.05374 (9)	0.0685 (6)	
H14A	−0.1895	1.0816	0.0595	0.103*	
H14B	−0.2820	1.0232	0.0434	0.103*	
H14C	−0.1892	0.9699	0.0240	0.103*	
C15	0.12116 (11)	1.02005 (14)	0.10272 (7)	0.0456 (4)	
H15A	0.1123	1.0684	0.0722	0.055*	
C16	0.20632 (11)	1.00669 (14)	0.12319 (7)	0.0447 (4)	
C17	0.22005 (12)	0.93221 (17)	0.17162 (8)	0.0529 (5)	
C18	0.28618 (11)	1.06494 (15)	0.09819 (7)	0.0461 (4)	
C19	0.37009 (12)	1.01170 (16)	0.09219 (8)	0.0565 (5)	
H19A	0.3776	0.9389	0.1060	0.068*	
C20	0.44247 (12)	1.06487 (18)	0.06604 (8)	0.0608 (5)	
H20A	0.4984	1.0286	0.0623	0.073*	
C21	0.43052 (12)	1.17234 (16)	0.04563 (7)	0.0538 (5)	
C22	0.34929 (13)	1.22816 (16)	0.05115 (8)	0.0578 (5)	
H22A	0.3425	1.3011	0.0373	0.069*	
C23	0.27740 (12)	1.17399 (16)	0.07773 (8)	0.0544 (5)	
H23A	0.2221	1.2115	0.0820	0.065*	
O4	0.29087 (9)	0.91451 (14)	0.19558 (6)	0.0807 (5)	

### Atomic displacement parameters (Å^2^)

**Table:**

	*U*^11^	*U*^22^	*U*^33^	*U*^12^	*U*^13^	*U*^23^
O1	0.0473 (7)	0.0605 (8)	0.0480 (7)	0.0011 (6)	−0.0026 (5)	0.0141 (6)
C1	0.0472 (9)	0.0418 (9)	0.0380 (9)	0.0019 (7)	0.0015 (7)	0.0018 (7)
C2	0.0442 (9)	0.0442 (9)	0.0390 (9)	0.0035 (7)	−0.0014 (7)	0.0002 (7)
N1	0.0655 (11)	0.0793 (14)	0.0680 (12)	−0.0223 (10)	0.0043 (9)	0.0057 (10)
O2	0.0982 (13)	0.1086 (15)	0.1132 (15)	−0.0251 (11)	0.0095 (10)	0.0500 (12)
C3	0.0530 (10)	0.0385 (9)	0.0387 (9)	0.0006 (7)	0.0008 (7)	−0.0009 (7)
O3	0.0677 (10)	0.1078 (14)	0.1556 (18)	−0.0113 (10)	0.0351 (11)	0.0174 (13)
C4	0.0496 (9)	0.0436 (10)	0.0445 (10)	−0.0030 (7)	0.0030 (7)	0.0015 (8)
C5	0.0468 (9)	0.0425 (9)	0.0405 (9)	0.0014 (7)	−0.0001 (7)	−0.0029 (8)
C6	0.0501 (10)	0.0452 (10)	0.0402 (9)	0.0048 (8)	−0.0018 (7)	0.0055 (7)
C7	0.0563 (10)	0.0488 (10)	0.0458 (10)	−0.0016 (8)	−0.0010 (8)	0.0084 (8)
C8	0.1032 (18)	0.0653 (14)	0.0898 (17)	0.0275 (13)	0.0165 (14)	0.0257 (13)
C9	0.0846 (14)	0.0847 (16)	0.0485 (12)	−0.0168 (12)	−0.0113 (10)	0.0076 (11)
C10	0.0800 (14)	0.0669 (13)	0.0676 (14)	−0.0178 (11)	−0.0094 (11)	0.0259 (11)
C11	0.0448 (9)	0.0492 (10)	0.0486 (10)	0.0028 (8)	−0.0006 (7)	0.0013 (8)
C12	0.0577 (12)	0.0658 (14)	0.0879 (16)	−0.0063 (10)	−0.0137 (10)	−0.0057 (12)
C13	0.0577 (11)	0.0726 (14)	0.0691 (13)	0.0102 (10)	0.0083 (10)	−0.0038 (11)
C14	0.0527 (10)	0.0902 (16)	0.0627 (13)	0.0075 (10)	−0.0050 (9)	0.0145 (12)
C15	0.0504 (10)	0.0468 (10)	0.0396 (9)	0.0006 (8)	0.0013 (7)	0.0060 (8)
C16	0.0455 (9)	0.0467 (10)	0.0420 (9)	−0.0014 (7)	0.0005 (7)	0.0019 (8)
C17	0.0458 (9)	0.0635 (12)	0.0494 (10)	−0.0002 (9)	−0.0016 (8)	0.0092 (9)
C18	0.0481 (9)	0.0507 (10)	0.0394 (9)	−0.0027 (8)	−0.0034 (7)	−0.0001 (8)
C19	0.0533 (10)	0.0557 (12)	0.0604 (12)	0.0012 (9)	0.0013 (9)	0.0089 (9)
C20	0.0484 (10)	0.0694 (13)	0.0646 (13)	0.0002 (9)	0.0032 (9)	0.0039 (10)
C21	0.0540 (10)	0.0600 (12)	0.0473 (10)	−0.0146 (9)	0.0005 (8)	−0.0002 (9)
C22	0.0650 (12)	0.0484 (11)	0.0600 (12)	−0.0077 (9)	−0.0025 (9)	0.0054 (9)
C23	0.0519 (10)	0.0514 (11)	0.0600 (11)	0.0008 (8)	−0.0009 (8)	0.0013 (9)
O4	0.0516 (8)	0.1155 (13)	0.0749 (10)	−0.0047 (8)	−0.0127 (7)	0.0394 (9)

### Geometric parameters (Å, °)

**Table:**

O1—C17	1.376 (2)	C11—C12	1.526 (3)
O1—C2	1.3804 (19)	C11—C14	1.527 (3)
C1—C2	1.391 (2)	C11—C13	1.531 (2)
C1—C6	1.398 (2)	C12—H12A	0.9600
C1—C15	1.431 (2)	C12—H12B	0.9600
C2—C3	1.398 (2)	C12—H12C	0.9600
N1—O3	1.216 (2)	C13—H13A	0.9600
N1—O2	1.216 (2)	C13—H13B	0.9600
N1—C21	1.466 (2)	C13—H13C	0.9600
C3—C4	1.390 (2)	C14—H14A	0.9600
C3—C7	1.534 (2)	C14—H14B	0.9600
C4—C5	1.405 (2)	C14—H14C	0.9600
C4—H4A	0.9300	C15—C16	1.347 (2)
C5—C6	1.373 (2)	C15—H15A	0.9300
C5—C11	1.528 (2)	C16—C17	1.459 (2)
C6—H6A	0.9300	C16—C18	1.481 (2)
C7—C9	1.528 (3)	C17—O4	1.200 (2)
C7—C8	1.529 (3)	C18—C23	1.387 (2)
C7—C10	1.533 (2)	C18—C19	1.389 (2)
C8—H8A	0.9600	C19—C20	1.379 (2)
C8—H8B	0.9600	C19—H19A	0.9300
C8—H8C	0.9600	C20—C21	1.374 (3)
C9—H9A	0.9600	C20—H20A	0.9300
C9—H9B	0.9600	C21—C22	1.368 (3)
C9—H9C	0.9600	C22—C23	1.384 (2)
C10—H10A	0.9600	C22—H22A	0.9300
C10—H10B	0.9600	C23—H23A	0.9300
C10—H10C	0.9600		
			
C17—O1—C2	123.84 (13)	C12—C11—C5	110.45 (15)
C2—C1—C6	119.39 (15)	C14—C11—C5	111.87 (14)
C2—C1—C15	118.41 (15)	C13—C11—C5	108.56 (14)
C6—C1—C15	122.20 (15)	C11—C12—H12A	109.5
O1—C2—C1	118.92 (14)	C11—C12—H12B	109.5
O1—C2—C3	118.49 (14)	H12A—C12—H12B	109.5
C1—C2—C3	122.59 (15)	C11—C12—H12C	109.5
O3—N1—O2	123.03 (19)	H12A—C12—H12C	109.5
O3—N1—C21	119.1 (2)	H12B—C12—H12C	109.5
O2—N1—C21	117.90 (19)	C11—C13—H13A	109.5
C4—C3—C2	114.90 (15)	C11—C13—H13B	109.5
C4—C3—C7	121.89 (15)	H13A—C13—H13B	109.5
C2—C3—C7	123.20 (14)	C11—C13—H13C	109.5
C3—C4—C5	124.96 (15)	H13A—C13—H13C	109.5
C3—C4—H4A	117.5	H13B—C13—H13C	109.5
C5—C4—H4A	117.5	C11—C14—H14A	109.5
C6—C5—C4	117.16 (15)	C11—C14—H14B	109.5
C6—C5—C11	122.55 (15)	H14A—C14—H14B	109.5
C4—C5—C11	120.24 (14)	C11—C14—H14C	109.5
C5—C6—C1	120.96 (15)	H14A—C14—H14C	109.5
C5—C6—H6A	119.5	H14B—C14—H14C	109.5
C1—C6—H6A	119.5	C16—C15—C1	122.66 (16)
C9—C7—C3	109.04 (15)	C16—C15—H15A	118.7
C9—C7—C8	110.76 (17)	C1—C15—H15A	118.7
C3—C7—C8	110.91 (15)	C15—C16—C17	118.68 (15)
C9—C7—C10	107.15 (16)	C15—C16—C18	122.24 (15)
C3—C7—C10	111.65 (14)	C17—C16—C18	119.07 (14)
C8—C7—C10	107.27 (16)	O4—C17—O1	116.10 (16)
C7—C8—H8A	109.5	O4—C17—C16	126.47 (17)
C7—C8—H8B	109.5	O1—C17—C16	117.43 (14)
H8A—C8—H8B	109.5	C23—C18—C19	118.07 (16)
C7—C8—H8C	109.5	C23—C18—C16	120.05 (15)
H8A—C8—H8C	109.5	C19—C18—C16	121.82 (16)
H8B—C8—H8C	109.5	C18—C19—C20	121.18 (18)
C7—C9—H9A	109.5	C18—C19—H19A	119.4
C7—C9—H9B	109.5	C20—C19—H19A	119.4
H9A—C9—H9B	109.5	C21—C20—C19	118.90 (18)
C7—C9—H9C	109.5	C21—C20—H20A	120.5
H9A—C9—H9C	109.5	C19—C20—H20A	120.5
H9B—C9—H9C	109.5	C22—C21—C20	121.77 (17)
C7—C10—H10A	109.5	C22—C21—N1	119.31 (18)
C7—C10—H10B	109.5	C20—C21—N1	118.91 (18)
H10A—C10—H10B	109.5	C21—C22—C23	118.69 (18)
C7—C10—H10C	109.5	C21—C22—H22A	120.7
H10A—C10—H10C	109.5	C23—C22—H22A	120.7
H10B—C10—H10C	109.5	C18—C23—C22	121.37 (17)
C12—C11—C14	108.27 (16)	C18—C23—H23A	119.3
C12—C11—C13	109.29 (16)	C22—C23—H23A	119.3
C14—C11—C13	108.35 (16)		

### Hydrogen-bond geometry (Å, °)

**Table:**

*D*—H···*A*	*D*—H	H···*A*	*D*···*A*	*D*—H···*A*
C6—H6A···O2^i^	0.93	2.55	3.409 (3)	154

Symmetry codes: (i) *x*−1/2, −*y*+5/2, −*z*.
